# The Natural History and Diseases of the Human Teeth

**Published:** 1847-01

**Authors:** 


ART. IV.—*The Natural History and Diseases of the human Teeth.* By JOSEPH FOX, M. R. C. S. L.; *Member of the Society of Medicine, Paris; Lecturer on the Structure and Diseases of the Teeth, at Guy's Hospital, and Surgeon Dentist to their Royal Highnesses, the Dukes of Kent and Sussex. First American from the third London Edition. Remodeled with an Introduction, and numerous additions.* By CHAPIN A. HARRIS, M. D., D. D. S., *Prof. of Practical Dentistry and Dental Pathology in the Baltimore College of Dental Surgery, etc. Illustrated with thirty Plates.* Philadelphia, Ed. Barrington & Geo. D. Haswell, 1846.

This work commences with an introduction by Prof Harris setting forth the importance of the subject of which it treats, and the ability, natural and acquired, which should characterize those engaging in this department of medical art. The *author* gives in chapter first, a description of the formation of the temporary set of teeth; in the second, of the formation of the permanent set of teeth; in the third, of the *manner* in which the teeth are formed; in the fourth, of the shedding of the teeth; in the fifth, of the irregularity of the teeth; in the sixth, of the treatment to *prevent* irregularity of the teeth; and in the seventh, of the treatment to *remedy* irregularity of the teeth. In the commencement of the last mentioned chapter the editor says: "There is no truth in surgery more fully established than is that of the practicability of altering the position of a tooth in the mouth, after the completion of its growth; and yet there is no branch of practice in dentistry more neglected than the treatment of irregularity in the arrangement of organs. Notwithstanding the acknowledged importance of regularity in the arrangement of the teeth, not only to an agreeable expression of countenance, but also to the health and durability of the whole dental apparatus,—hardly one practitioner in twenty ever gives the subject a thought. Their manipulations are almost wholly confined to filing and plugging the natural teeth, and the substitution of artificial ones for their loss. The attention of a few of the more scientific and skilful practitioners, however, has been directed to the treatment of *irregularity*, and the results of their labors, in this department of physical alleviation, have been as gratifying to their own feelings as they have been beneficial to their patients."—Page 87. And again, in chapter ninth, on the the subject of the decay of the temporary teeth, the editor says: "The preservation of the temporary teeth until they are removed by the operations of the economy, to give place to the permanent ones, or until the latter are about to appear, is of the utmost importance, and this, in a majority of cases, might be effected by timely and proper attention. The temporary teeth should be cleaned with a brush and waxed floss silk three or four times every day, and if this were done from the time they make their appearance, there would not be one decayed deciduous tooth where there are now twenty. Sound teeth are as desirable and just as necessary to the health and comfort of a child as they are to an adult, and therefore they should not be permitted, from neglect of the means of their preservation, to decay; and the temporary teeth require as much care as do the permanent ones, and they should never be extracted except for the relief of pain that cannot be relieved by any other means, or the cure of an alveolar abscess, or to make room for a permanent tooth. The popular opinion that inasmuch as the teeth are to be replaced with others, it is of little importance whether they remain in the mouth until they are removed by nature, to make room for their successors, or are lost a year or two earlier, is erroneous, and has been productive of much injury."—Pages 106 and 107.

In speaking of caries the author says: "That the teeth acquire a disposition to *decay*, from some want of healthy action during their formation, seems to be proved by the common observation that they become decayed in pairs; that is, those teeth which are formed at the same time, being in a similar state of imperfection, have not the power to resist the causes of disease, and, therefore, nearly about the same period, they exhibit signs of decay, while those teeth which have been formed at another time, when a more healthy action existed, have remained perfectly sound to the end of life."—Page 167. Again the editor adds: "*Temperament* and the state of the constitutional health at the time of the ossification of the teeth, have much to do in determining their susceptibility to the action of the causes that produce decay, and these are influenced in a great degree by residence in certain localities, and modes of living."—Page 175.

We have made these quotations for two reasons; 1st, on account of their value; and 2d, to give the reader a sample of the spirit and style of the whole work. Prof. Harris has in this work accomplished two objects, 1st, the clear exhibition of the great improvements in dentistry in the last quarter century; 2d, the redemption from oblivion of a standard work of the past, the production of one of the most learned and zealous promotors of medical science, by presenting it in a dress modern and agreeable.

The remainder of the second part is taken up with a description of the treatment of other diseases of the teeth, diseases of the gums and alveolar processes, &c.; and of the operations required in accomplishing the relief which is available through this department of medical art.

Part third closes the volume, with a clear yet concise description of artificial teeth, obturators and palates, the indications for their use, directions for their application, and the benefits following their discriminate employment. We have read the volume with profit and pleasure, and recommend it most cordially to our readers.

